# Determinants of poor self-rated health among adults in urban Mozambique

**DOI:** 10.1186/s12889-016-3552-5

**Published:** 2016-08-24

**Authors:** Boaventura M. Cau, Joana Falcão, Carlos Arnaldo

**Affiliations:** 1 Departamento de Geografia, Universidade Eduardo Mondlane (UEM), C.P. 257 Maputo, Mozambique; 2 Centro de Pesquisa em População e Saúde (CEPSA), Maputo, Mozambique

**Keywords:** Determinants, Self-rated health, Poor health, Adults, Urban areas, Mozambique

## Abstract

**Background:**

Self-rated health is a measure expressing the general condition of health of individuals. Self-rated health studies are common in developed countries and in some developing regions. Despite increasing proportion of adult and older population in sub-Saharan Africa and poor population health indicators, there is a dearth of studies on self-rated health in the region. This study examines factors associated with poor self-rated health among adult individuals in Maputo metropolitan area in Mozambique.

**Methods:**

Data for this study come from a survey of 1768 individuals aged 18 years or more carried out in Maputo metropolitan area, Mozambique, in 2015. Employing multiple logistic regression, the study used a subsample of 677 female and male respondents aged 40 years or more to estimate the determinants of poor self-rated health.

**Results:**

About 54 % of respondents aged 40 years or more believed that their health status was poor. Female respondents [Odds Ratios (OR) = 3.43, *p* <0.01], single (OR = 4.71, *p* < 0.05), widow (OR = 1.81, *p* < 0.05), separated or divorced (OR = 2.08, *p* < 0.05) and those believing that hypertension or heart problem was a major community health problem (OR = 1.56, *p* < 0.05) displayed higher odds of reporting poor health than their peers, net of other factors. Furthermore, individuals aged 40–49 years (OR = 0.45, *p* < 0.01), or 50–59 years (OR = 0.59, *p* < 0.05), those whose work involves intensive physical activity (OR = 0.60, *p* < 0.05) and those from households treating drinking water (OR = 0.49, *p* < 0.01) showed lower odds of reporting poor health, adjusting for other factors.

**Conclusion:**

Overall, the results point to the importance of age, gender, marital status, socioeconomic circumstances, individuals’ health behaviors and perceived community health problems as key determinants of poor self-rated health among adults in Maputo metropolitan area. Given the growing number of adult and older people in sub-Saharan Africa, the rising importance of non-communicable diseases and the scarcity of studies on determinants of poor self-rated health among adults in the region, our findings may have implications for a better understanding of the drivers of poor health among adults in urban sub-Saharan Africa

## Background

Self-rated health is a measure expressing the general condition of health of individuals [1] and it has been recommended by the World Health Organization as an indicator of the health status of older individuals [2]. Self-rated health studies are common in developed countries [3–5] and in some developing countries [6–11]. Research on self-rated health in developed countries finds that self-rated health predicts functional decline [12], chronic health disorders [13, 14] and mortality [15, 16]. According to a recent study, sub-Saharan Africa is the region of the world where the number of people aged 60 years or more is growing rapidly [17]. The number of people aged 60 years or more in sub-Saharan Africa was estimated at 34 million in 2005 and it is projected that in 2030 it will be over 67 million [17]. Despite increasing proportion of adult and older population in sub-Saharan Africa [18] and poor population health indicators, [19] there is a dearth of studies on self-rated health in the region.

Although there are some studies on self-rated health in sub-Saharan Africa [11, 20–24] there is still a limited understanding of the determinants of poor self-rated health among adults and older people in the region. In this study we investigate factors associated with poor self-rated health among adults in Maputo metropolitan area in Mozambique. In particular, we investigate the role of demographic and socioeconomic factors, individual health behaviors and individuals’ perceptions of community main health problems. Like most other countries of sub-Saharan Africa, Mozambique is a country with a growing size of older population especially in urban areas [25] and poor health indicators, [19] which makes it a relevant place to study determinants of poor self-rated health.

Numerous previous studies in developed countries and a few in developing countries have suggested possible correlates of poor self-rated health. Considering the age of individuals, previous studies have indicated that older persons are more likely to report poor self-rated health compared to younger ones [10, 26]. Most studies also find that women are more likely to report poor self-rated health than men [10, 26]. As for the connection between marital status and poor self-rated health, some studies suggested that divorced, separated or widowed were more likely to report poor health than married or never married individuals [26, 27]. Also a study suggested that those living alone were more likely to report poor health compared to those who were not [28].

With respect to the role of education, studies typically find that those with more years of education tend to report better self-rated health compared to those with lower years of education [10, 29]. A link between employment and self-rated health has also been suggested. For example, a study in South Africa noted that unemployment contributed for poor self-rated health [30]. Studies in developed countries have suggested that holding a high prestigious job was associated with lower odds of reporting poor self-rated health [31]. Household sanitation and household size in developing countries also appear to be important correlates of self-rated health. For example, a study in Kenya found that the majority of those from households using public flush toilet, pit latrines and those from households having unreliable access to water reported poor self-rated health [11]. Connections between health behaviors and self-rated health also have been suggested. Studies in developed countries indicated that physically inactive [32, 33] underweight and obese people [32] displayed a high risk of having poor self-rated health than their counterpart.

Individuals’ perceptions of community health problems may be associated with poor self-rated health as well [34]. Many authors have argued that community health circumstances (e.g., prevalence of crime, environmental sanitation and general hygiene in neighborhoods) may have effects on health outcomes [11, 35–37]. For example, a study argued that “…fear of crime can indirectly affect health by increasing stress, promoting social isolation, preventing the health promoting practices of walking for exercise, and preventing access to services for those fearful of travelling freely in the community.” [35] These circumstances could ultimately contribute for poor self-rated health. Although the evidence is scarce, it is also possible that perceptions of community health conditions (e.g., perceptions of prevalence of malaria, HIV/AIDS or people with hypertension in the community) could be connected with poor self-rated health. Our study attempts to contribute for filling the gap in the existing literature about the main drivers of poor self-rated health among adults in sub-Saharan Africa. Identifying key factors that increase the risk of poor health among adults may be useful for designing appropriate interventions for improving the health of this population group.

## Methods

This study uses data from the “Health Barometer: Individual and Community Health Promoting Practices in Maputo City” project. This project was carried out by the Center for Research on Population and Health (*Centro de Pesquisa em População e Saúde* – CEPSA) in Maputo, Mozambique. The purpose of the research project was to examine individual and community health promoting knowledge, attitudes and practices and to assess public perceptions about accessibility and quality of health services provided in Maputo metropolitan area. The study was authorized by the National Bioethical Committee for Health in Mozambique (IRB00002557). Consent was obtained from all participants in the study. Data was collected in March 2015.

The sample of the study was probabilistic and representative of the population of continental Maputo metropolitan area (excluding Ka Tembe and Ka Nyaka island). The sampling was based on the Mozambique’s 2007 Census sample frame for Maputo City. The sample was selected in three stages. First, 50 enumeration areas (neighborhoods) were randomly selected within 7 municipalities of Maputo metropolitan area. Second, in each enumeration area, 22 households were randomly selected from the list of all households. Finally, in each of the selected households, one female and one male individual were selected for interview if there were 2 or more adult individuals in the household. If there was only one adult individual (female or male) in the selected household, the interview was held with the existing adult. Overall, the study collected data on 1768 individuals aged 18 years or more. The response rate in the study was estimated in 83.9 % [38]. The study collected data on several topics including sociodemographic characteristics, household characteristics, perceptions about health care services, perceptions about own health and perceptions about community health (further details may be obtained in the main survey report [38]). In the overall study sample, there were 686 female and male individuals aged 40 years or more. Of the 686 individuals, 9 had missing information on variables of interest, resulting in an analytic sub-sample of 677 individuals.

### Measures

Respondents were asked to indicate the status of their own health at the time of the interview. Possible responses could be “excellent”, “very good”, “good”, “fair”, “bad” and “very bad”. Answers to the question were used to create a measure of self-rated health with two categories – good health (good, very good or excellent) versus poor health (fair, bad or very bad). This is the main outcome variable. Our predictor variables of interest attempt to measure (i) demographic and socioeconomic factors, (ii) individuals’ health behaviors, and (iii) individuals’ perceptions about main community health problems.

The demographic and socioeconomic factors considered are individuals’ age, sex, marital status, education, type of occupation, type of toilet used in the household, whether or not the respondent’s household treats drinking water, whether or not the respondent’s household owns a car and the household family size. Age has three categories: 40–49 years, 50–59 years and 60 years or more – the reference. Sex has two categories – female and male (reference). Marital status categories are single, divorced or separated, widow and married (or in marital union), which is the reference. Education of the respondent was divided into no education, primary education, and secondary education or more (reference). Respondent’s occupation type has seven categories: domestic work, professional work (reference), business work, small sells work, art work, student or unemployed, and other occupation. The type of toilet used in the household has four categories which are: flush toilet (reference), non-flush toilet, improved latrine, non-improved latrine or no latrine in the residence. Whether or not the respondent’s household treats drinking water has two categories – drinks treated water (e.g., boiled water, mineral water, filtered water or treated water) and does not drink treated water (reference). Household’s ownership of a car has two categories – owns a car (reference) and does not own a car. Household size is a continuous variable indicating the total number of people in the household.

To measure individuals’ health behaviors we considered perceived body weight (a proxy for individual behaviors), practice of physical activity, smoking, alcohol consumption. For getting perceived-weight, respondents were asked to say how they consider their weight to be, taking into account their age and height. Categories of perceived body weight were high or overweight (reference), normal weight, low weight, and don’t know. With respect to physical activity, respondents were asked whether their work involves intensive physical activity that causes an increase in frequency of the heart beat for at least continuous 10 min. This variable has two categories which are: yes or no (reference). Respondent’s smoking status has two categories – yes or no (reference). Respondent’s alcohol consumption has four categories, which are: no alcohol (reference), monthly or less, 2–4 times per month, 2 times or more per week.

Respondents were also asked to name three main health problems in their community. Answers to this question were used to create ten variables measuring perceived main community health problems which are: sanitation problem (yes vs. no – reference); alcohol consumption (yes vs. no – reference); tobacco consumption (yes vs. no – reference); drugs’ consumption (yes vs. no – reference); crime (yes vs. no – reference); road accidents (yes vs. no – reference); respiratory problems (yes vs. no – reference); hypertension or heart problems (yes vs. no – reference); HIV/AIDS problem (yes vs. no – reference), and malaria, tuberculosis or diarrhea problem (yes vs. no –reference). Table 1 shows descriptive statistics of the study sample.

**Table 1:** Descriptive Statistics of the study sample (percent unless indicated), Health Barometer, Maputo Metropolitan Area, Mozambique, 2015

Variable	Percent
Poor Self-rated Health	
No	46.2
Yes	53.8
*Demographic and Socioeconomic Factors*	
Age	
40-49	41.3
50-59	34.2
60 or more	24.5
Sex	
Male	45.4
Woman	54.6
Marital Status	
Married or in union	71.4
Single	1.9
Separated or divorced	9.7
Widow	17.0
Education	
No education	31.6
Primary	41.8
Secondary or more	26.6
Type of Occupation	
Professional work	27.0
Domestic work	16.1
Business work	16.1
Small sells	12.2
Art work	6.7
Unemployed or student	7.2
Other occupation	14.7
Type of Toilet	
Flush toilet	30.1
Non-flush toilet	37.8
Improved latrine	23.8
Non-improved latrine or no latrine in the residence	8.3
Drinking water treatment	
Does not treat	71.3
Treats	28.7
Household car ownership	
Does not own a car	75.7
Owns a car	24.3
Family size (mean, standard deviations in parenthesis)	6.0 (2.8)
*Health Behaviors*	
Perception about own body weight	
High or overweight	26.6
Normal	54.2
Low body weight	16.1
Don't Know	3.1
Work involves intensive physical activity	
No	70.4
Yes	29.6
Smoking status	
No	91.0
Yes	9.0
Alcohol consumption	
No alcohol	50.9
Monthly or less	24.5
2-4 times per month	14.1
2 times or more per week	10.5
*Perceptions about Community Main Health Problems*	
Sanitation problem	
No	92.0
Yes	8.0
Alcohol consumption	
No	50.2
Yes	49.8
Tobacco consumption	
No	90.0
Yes	10.0
Drugs’ consumption	
No	91.0
Yes	9.0
Crime	
No	95.2
Yes	4.8
Road accidents	
No	95.7
Yes	4.3
Respiratory problems	
No	86.8
Yes	13.2
Hypertension or heart problem	
No	56.3
Yes	43.7
HIV/AIDS problem	
No	60.6
Yes	39.4
Malaria, tuberculosis or diarrhea	
No	59.1
Yes	40.9
N	677

### Statistical analysis

Data was prepared and analyzed using Stata version 11 [39]. Our models were estimated using multiple logistic regression. Model 1 examines effects of demographic and socioeconomic factors. Model 2 assesses effects of individuals’ health behaviors. Model 3 adds demographic and socioeconomic factors as controls. Model 4 examines effects of individuals’ perceptions about main community health problems. Model 5 adds demographic and socioeconomic factors. Model 6 is a full model assessing effects of all predictors considered in the present study. In the analysis, weights were included to take into account the complex survey design.

## Results

**Fig. 1:**
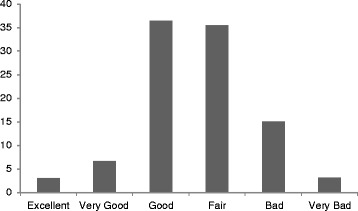
Distribution of respondents by items of self-rated health status

Figure 1 shows the distribution of respondents by items of self-rated health. Nearly a similar proportion of respondents rated the status of their health as good (36.4 %) or fair (35.5 %). About 15 % of respondents believed that their health status was bad and only about 3 % rated their health status as excellent (Fig. 1). Overall, of the 677 respondents in the sample, approximately 54 % believed that their health status was poor (fair, bad or very bad), 55 % were female, almost 71 % were married or in marital union, 32 % had no education (Table 1).

Table 2 presents logistic regression results of determinants of poor self-rated health among adults in Maputo metropolitan area. The results are shown as Odds Ratios. Model 1 has demographic and socioeconomic factors as predictors. The results in Model 1 show that poor self-rated health significantly increases with age. Compared to men, women display higher odds of reporting poor self-rated health (*p* < 0.01). Relative to married (or in marital union), individuals who are single, widow, separated or divorced significantly display worse self-rated health. Model 1 also shows that individuals with primary education have significantly higher odds of reporting poor self-rated health than those with secondary education or more. It also shows that those engaged in small sells’ activities and those from households treating drinking water significantly have better self-reported health than their references.

**Table 2:** Logistic regression results of determinants of poor self-rated health among adults, Health Barometer, Maputo Metropolitan Area, Mozambique, 2015 (Odds Ratio)

Variable	Model 1	Model 2	Model 3	Model 4	Model 5	Model 6
*Demographic and Socioeconomic Factors*						
Age						
60 or more (reference)	1		1		1	1
40-49	0.41**		0.45**		0.41**	0.45**
50-59	0.53*		0.57*		0.56*	0.59*
Sex						
Male (reference)	1		1		1	1
Woman	3.14**		3.31**		3.23**	3.43**
Marital Status						
Married or in union (reference)	1		1		1	1
Single	3.56*		3.80*		4.27*	4.71
Separated or divorced	2.00*		2.02*		2.06*	2.08*
Widow	1.84*		1.80*		1.86*	1.81*
Education						
Secondary or more (reference)	1		1		1	1
No education	0.87		0.78		0.83	0.74
Primary	1.85*		1.71*		1.76*	1.64+
Type of Occupation						
Professional work (reference)	1		1		1	1
Domestic work	0.62		0.55+		0.60	0.52+
Business work	0.80		0.77		0.78	0.75
Small sells	0.26**		0.25**		0.25**	0.23**
Art work	0.82		0.84		0.86	0.88
Unemployed or student	0.95		0.85		0.92	0.81
Other occupation	0.92		0.93		0.89	0.90
Type of Toilet						
Flush toilet (reference)	1		1		1	1
Non-flush toilet	1.21		1.27		1.17	1.23
Improved latrine	0.71		0.71		0.72	0.73
Non-improved latrine or no latrine in the residence	1.32		1.39		1.28	1.36
Drinking water treatment						
Does not treat (reference)	1		1		1	1
Treats	0.46**		0.46**		0.49**	0.49**
Household car ownship						
Does not own a car (reference)	1		1		1	1
Owns a car	1.00		1.01		0.97	0.99
Family size	1.02		1.02		1.03	1.02
*Health Behaviors*						
Perception about own body weight						
High or overweight (reference)		1	1			1
Normal		1.09	1.22			1.22
Low body weight		1.54	1.63			1.70+
Don't know		1.36	1.69			1.92
Work involves intensive physical activity						
No (reference)		1	1			1
Yes		0.63*	0.62*			0.60*
Smoking status						
No (reference)		1	1			1
Yes		0.71	0.76			0.82
Alcohol consumption						
No alcohol (reference)		1	1			1
Monthly or less		1.01	0.99			0.98
2-4 times per month		0.62+	0.82			0.80
2 times or more per week		0.78	1.20			1.15
*Perceptions about Community Main Health Problems*						
Sanitation problem						
No (reference)				1	1	1
Yes				1.06	1.17	1.21
Alcohol consumption						
No (reference)				1	1	1
Yes				1.01	1.01	1.01
Tobacco consumption						
No (reference)				1	1	1
Yes				0.88	0.81	0.77
Drugs’ consumption						
No (reference)				1	1	1
Yes				1.43	1.31	1.32
Crime						
No (reference)				1	1	1
Yes				0.45*	0.40*	0.42*
Road accidents						
No (reference)				1	1	1
Yes				0.89	0.88	0.94
Respiratory problems						
No (reference)				1	1	1
Yes				0.62	0.69	0.69
Hypertension or heart problem						
No (reference)				1	1	1
Yes				1.71**	1.45+	1.56*
HIV/AIDS problem						
No (reference)				1	1	1
Yes				1.31	1.24	1.26
Malaria, tuberculosis or diarrhea						
No (reference)				1	1	1
Yes				1.12	1.07	1.00
Intercept	1.12	1.34	1.09	0.86	0.92	0.86
Model Chi-Square	86.86**	15.22+	94.92**	16.74+	94.25**	97.03**
N	677	677	677	677	677	677

+ − *p* < 0.1; *- *p* ≤ 0.05; **- *p* ≤ 0.01

Model 2 assesses effects of individuals’ health behaviors on poor self-reported health. The results suggest that those whose work involves physical activity show lower odds of reporting poor self-rated health than their peers [Odds Ratios (OR) = 0.63, *p* <0.01]. Model 3 controls for demographic and socioeconomic factors and it finds that the association observed in Model 2 remains strong.

Model 4 examines associations between individuals’ perceptions about main community health problems and poor self-rated health. Surprisingly, the results suggest that believing that crime is the main community health problem is negatively associated with reporting poor self-rated health. The results also show that the odds of reporting poor self-rated health for individuals who believe that hypertension or heart problem is the main community health problem are 71 % higher than the reference group. Model 5 adds demographic and socioeconomic factors. The negative association of perception of crime in the community and poor self-related health remains strong but the association between perceptions of hypertension or heart problem as the main community health problem and poor self-rated health diminishes.

Model 6 examines the effect of all determinants of poor self-rated health considered in this study. Model 6 shows that being aged 60 years or more, woman, single, widow, separated or divorced, and believing that hypertension or heart problem is the main community health problem is significantly associated with reporting poor self-assed health net of other factors. Model 6 also indicates that individuals engaged in small sells, those from households treating drinking water, those whose work involves intensive physical activity, and those believing that crime is the main community health problem, significantly show lower odds of reporting poor self-rated health adjusting for other factors.

## Discussion

Although research on self-rated health is well established in developed countries and some developing countries, in sub-Saharan Africa there is still a limited understanding of key determinants of poor self-reported health. The analysis we presented above attempted to examine the role of demographic and socioeconomic factors, individual health behaviors and individuals’ perceptions of community main health problems, on poor self-rated health among adults in urban Mozambique. Age, gender and marital status were found to be key determinants of poor self-rated health. Consistent with previous studies, as the individual’s age increases the likelihood for reporting poor self-rated health also increases [10]. Also in line with past studies, women show poor self-reported health than male individuals [29]. Single, widow, separated or divorced displayed significantly poor self-rated health compared to married (or in marital union) individuals [29, 40]. It seems that being married offers a buffer for poor health possibly through greater access to social support and other resources that marriage offers [41].

This study also found that those whose employment involves small sells displayed better self-rated health than those in professional occupations. On the one hand, this finding may reflect differential socioeconomic circumstances as some of those involved in small sells (e.g., informal trade) are economically better off than most of those in low paying professional jobs. As Peberdy and colleagues have noted [42, 43], although the informal sector is typically characterized as marginal, in big cities of southern Africa such as Maputo, some informal traders have high incomes. On the other hand, it is also possible that the finding that those involved in small sells displayed lower odds of poor self-rated health than those in professional occupations may reflect the tendency of different population groups to attach different meanings to the ratings of health [44]. The study also found that individuals from households treating drinking water showed lower odds of reporting poor self-rated health than their peers. It is likely that those whose households treat drinking water may be from affluent households that typically have many resources and large network support that may act in ways that are favorable to health [45]. With respect to the role of individuals’ health behaviors, findings from the present study corroborate evidence from previous studies [32, 33]. Results indicate that those whose work involves intensive physical activity reported good health than their peers. This finding highlights the importance of encouraging physical activity as a mean of promoting health.

In this study we argued that perceptions of community health problems could be linked with poor self-rated health as such perceptions often stem from existing socioeconomic and health circumstances in the community. Perceptions of community health problems may act in ways that undermine individuals’ health through psychosocial processes [46, 47]. In the last model, which considered all variables, we found that those who believed that hypertension or heart problem is the major health problem affecting the community displayed higher odds of reporting poor health than their counterpart net of other factors.

In this study, we also unexpectedly found that the odds of reporting poor self-rated health among those thinking that crime is the main problem afflicting their community were lower than those of their peers net of demographic and socioeconomic factors. This unexpected finding may be related to a differential perception of crime prevalence between poor people and affluent ones. If affluent people perceive crime as highly prevalent in the community compared with poor people, a negative association between perception of crime prevalence and poor health could occur. In fact, in the last three to four years, in Maputo metropolitan area there has been a new type of crime characterized by the kidnappings of affluent people [48] which is likely to be less felt by poor people.

This study has limitations. It is possible that those who perceived hypertension and heart problem being a community problem could be those who had been diagnosed with such a problem. If they have been diagnosed with hypertension or a heart problem they could be more aware of such a problem in the community. However, with our data we are unable to verify such a possibility, as the questionnaire did not ask respondents whether they have been diagnosed with hypertension or a heart problem. In the questionnaire, there was a question asking whether respondents have ever used health care services that could have been used as control. Nonetheless, almost all respondents in the subsample replied affirmatively to this question.

The fact that our independent variables are self-reported and that our survey was not powered specifically for this analysis are another important potential limitations of the present study. We considered the possibility of conducting multilevel analysis. Yet, because of the small analytic sample size and the number of predictors of interest, the number of cases in neighborhoods for some predictors of interest was not sufficient for obtaining unbiased estimates. In addition, it should be noted that comparing self-rated health across subgroups (e.g., as defined by gender or age) may be subjected to the different cut-offs that subgroups could place between one category and the other (e.g., what a group might consider fair health could be very good health for another group due to different expectations). Despite these limitations, our findings may have implications for a better understanding of the drivers of poor health among adults in Maputo metropolitan area and similar settings of urban sub-Saharan Africa.

The Ministry of Health of Mozambique has just approved a National Health Promotion Strategy (2015–2024) [49], which is based on a multi-sectorial approach together with strong community engagement strategies to prevent a range of communicable and non-communicable diseases. The results of the present study can be used as a baseline of the current situation in Maputo metropolitan area for some of the drivers of poor health examined in this study. In addition, this study, by contributing to the understanding of the factors that underlie self-rated health in Maputo metropolitan area, may be used as evidence to support the designing and implementation of future health promotion programs in the city.

## Conclusion

The analysis we presented in this study points to the importance of age, gender, marital status, socioeconomic circumstances, individuals’ health behaviors and perceived community health problems as key determinants of poor self-rated health in Maputo metropolitan area. Given the growing number of adult and older people in sub-Saharan Africa [17, 18] and the rising importance of non-communicable diseases such as stroke and diabetes in the region [50] more studies that may lead to a better understanding of determinants of poor self-rated health among adults in sub-Saharan Africa are still needed. Our study attempted to contribute for filling the gap of knowledge on this important population health issue in sub-Saharan Africa.
